# An Arterial Spin Labeling MRI Perfusion Study of Migraine without Aura Attacks

**DOI:** 10.3389/fneur.2017.00280

**Published:** 2017-06-28

**Authors:** Raquel Gil-Gouveia, Joana Pinto, Patricia Figueiredo, Pedro Ferro Vilela, Isabel Pavão Martins

**Affiliations:** ^1^Headache Center, Hospital da Luz, Lisboa, Portugal; ^2^Institute for Systems and Robotics - Lisboa and Department of Bioengineering, Instituto Superior Técnico, Universidade de Lisboa, Lisbon, Portugal; ^3^Neurorradiology Department, Hospital da Luz, Lisboa, Portugal; ^4^Department of Clinical Neurosciences, Faculdade de Medicina, Universidade de Lisboa, Lisbon, Portugal

**Keywords:** migraine, cerebrovascular circulation, magnetic resonance imaging, perfusion arterial spin labeling neuroimaging

## Abstract

**Background:**

Studies of brain perfusion during migraine without aura attacks have inconsistent results.

**Methods:**

Arterial spin labeling MRI, a non-invasive quantitative perfusion technique, was used to prospectively study a spontaneous untreated migraine without aura attack and a headache-free period. Image analysis used FSL and MATLAB software; Group analysis used permutation methods for perfusion differences between sessions.

**Results:**

Thirteen women (age 35.7) were scanned during an attack of an average intensity of 6.8 (on 0–10 Visual Analog Scale) and 16 h duration. No global or regional perfusion differences were identified when comparing migraine and migraine-free sessions.

**Discussion:**

Our findings suggest that the painful phase of migraine without aura attacks is not associated with brain perfusion abnormalities.

## Introduction

Initial studies of brain perfusion during migraine attacks in the 70s measuring regional cerebral blood flow (CBF) with ^133^Xe identified a biphasic pattern of hypoperfusion during the aura and hyperperfusion during headache (1), suggesting the existence of different perfusion patterns in the aura and headache phase of the attacks. The visual aura has since then been consistently associated with a posteroanterior spreading wave of initial transient hyperperfusion followed by sustained hypoperfusion (CBF variation of −10 to −35%) (2) demonstrated in human subjects by different techniques [^133^Xe, SPECT, PET, and perfusion weighted imaging (PWI)-MRI (3)]. It is nowadays widely accepted that CBF changes occurring during the aura are associated with cortical spreading depression-like phenomena (4).

However, these findings seem to be related to the symptom of the aura. In migraine attacks with and without aura, the headache symptom itself did not seem to be related to brain perfusion changes (2, 5–7). These differences led to the separation of migraine into two nosological entities of migraine (with and without aura) in the International Classification of Headache Disorders (ICHD) (4, 8).

These assumptions were made based on low-quality data, as most of these studies were done in the 80s with ^133^Xe and Xe-SPECT (5, 6, 9–12), and only some (5, 6, 10, 11) separately evaluated patients with migraine without aura. Three studies of brain perfusion in migraine without aura using more recent and reliable techniques were published, two in the 90s, that revealed dissonant findings—one included nine patients studied with PET and demonstrated slight CBF reduction during a migraine without aura attacks (7), while a (PWI-MRI) study of 13 subjects was negative (2). A third later study in 2008 used PET to scan seven patients and was able to identify a regional bilateral posterior hypoperfusion in five patients, which persisted after successful headache treatment with sumatriptan (13).

In view of the scarce data available, we aimed to revisit this controversial issue and performed a controlled study with repeated measurements of global and regional CBF during attacks of migraine without aura and in an headache-free period using a non-invasive quantitative highly reliable MRI perfusion technique, arterial spin labeling (ASL-MRI) (14).

## Materials and Methods

### Population

Otherwise healthy adults (20–45 years) with ICHD 3-beta episodic migraine without aura were recruited among hospital staff and in the acute care outpatient clinic. The presence of aura, other headache types, and chronic migraine were exclusion criteria, as were claustrophobia and any contraindication to perform MRI. To minimize the effects of pharmacological agents, the only allowed medication was oral contraception. There was no financial compensation for the volunteers. This study was carried out in accordance with the recommendations of Comissão de Ética para a Investigação Clínica with written informed consent from all subjects. All subjects gave written informed consent in accordance with the Declaration of Helsinki. The protocol was approved by the Hospital da Luz Ethics Committee.

### Study Design

Prospective longitudinal study evaluating the same subject in two conditions, first during a spontaneously occurring migraine without aura attack and also in a headache-free period, at least a month later, and with a minimal 48 h delay from the last attack. No migraine relief medication was allowed 12 h preceding the attack MRI that required a minimal headache intensity of 4 on a 0–10 Visual Analog Scale (VAS) and the absence of pain intensity improvement in the hour preceding the evaluation. *Post hoc* analysis compared the characteristics of patients scanned in the early (≤5 h) and late phases of the attack. Both evaluations included a brief interview preceding the scan, which included collecting clinical data and migraine impact [headache impact test (HIT)-6 (15)].

### Image Acquisition

Volunteers were studied on 3-T Siemens Verio MRI system (Siemens, Erlangen, Germany) using a 12-channel head RF coil. Subject’s motion was restricted with foam padding between the head and the coil. For each subject, a T1-weighted structural image (3D T1 MPRAGE, TR = 2,250 ms, TE = 2.26 ms, voxel size of 1 mm × 1 mm × 1 mm) and PASL (Q2TIPS technique; PICORE labeling scheme; 2D echo planar-GE-EPI-readout, TR = 2,500 ms, TE = 11 ms, TI1 = 700 ms, TIs = 1,600 ms, TI2 = 1,800 ms, with nine contiguous axial 8 mm thickness slices with a voxel resolution of 4 mm × 4 mm × 10 mm were acquired in an ascending order headache-free images) were obtained during rest.

### Image Processing and Analysis

Image analysis was performed using FSL (http://fsl.fmrib.ox.ac.uk/) (16) and MATLAB custom based tools (The MathWorks, Inc., USA). ASL data preprocessing included brain extraction (17), motion correction (18), temporal filtering with a 100 s frequency cutoff, and spatial smoothing with a 5 mm full-width half maximum Gaussian kernel. Data were also co-registered to an expanded functional image, a main structural image, and a standard space using the FSL tool FLIRT (18). Subsequently, control and labeled images were pairwise subtracted, and perfusion weighted maps were computed by normalization with the brain equilibrium magnetization estimated from the averaged control images. Ten regions of interest (ROI) were identified according to the MNI152 atlas (McConnell Brain Imaging Center, Montreal Neurological Institute, McGill University, Montreal, QC, Canada), and group averaged CBF values were assessed for each ROI and session.

### Statistical Analyses

Statistical analysis of clinical variables was carried out using SPSS 20.0.

Descriptive statistics are presented as absolute and frequencies or mean ± SD; clinical characteristics were compared between groups with Student’s *t*-test. The primary study endpoint was to determine total and regional CBF variation between headache-free and the attack. CBF variation was calculated by subtracting the total CBF on the headache-free session from the total CBF of the migraine attack (attack CBF–headache-free CBF = ΔCBF) and by averaging the ΔCBF to the headache-free CBF (ΔCBF/headache-free CBF = ΔCBF%). The secondary endpoint was to determine if any independent variable, either clinical (age, literacy, disease duration, HIT-6, and time between evaluations) or attack-related (pain duration and intensity, nausea, photophobia, phonophobia, and aggravation with physical effort) had influence on ΔCBF, which was considered as the dependent variable on a multiple linear regression analysis. Type I error was set at the two-sided 1% significance level.

## Results

### Population

Fourteen right-handed female patients were included; one dropped out retaining 13 subjects with an age average of 35.7 ± 7.4 years, 6 (46%) used oral contraception. Six volunteers were mild smokers (less than 10 U a day), yet none had smoked at least 30 min before each scan. Average Beck score was 5.0 ± 3.6; all subjects had normal values. Average migraine history lasted for 22.7 ± 10.2 years; HIT-6 score was 62 ± 4.0 (high impact disease). Average monthly attack frequency in the 3 months preceding the study was 2.3 ± 1.6 (ranging from 1 to 6), with an average attack duration of 32.6 ± 25.3 h and an average intensity of 7.4 ± 1.3 on a 0–10 VAS scale.

### Migraine Attack Evaluation and Comparison with Headache-Free

**Figure 1:**
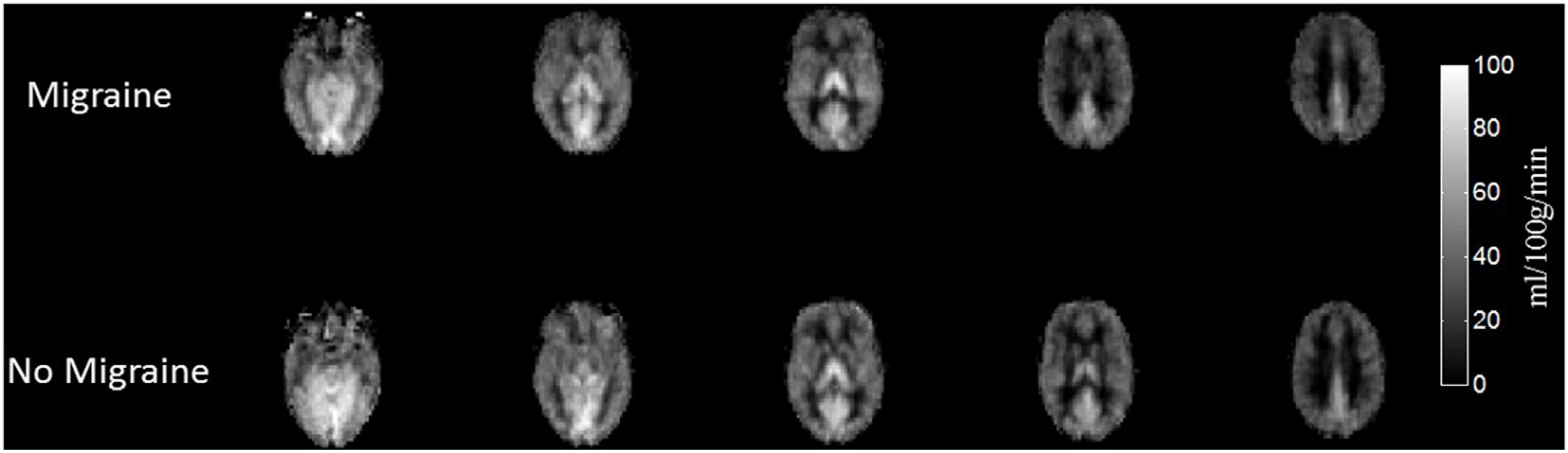
Cerebral blood flow (CBF) maps averaged across subjects during the migraine attack and attack-free status. CBF maps averaged across subjects in both conditions (migraine attack and attack-free status) for six representative slices in MNI standard space.

**Figure 2:**
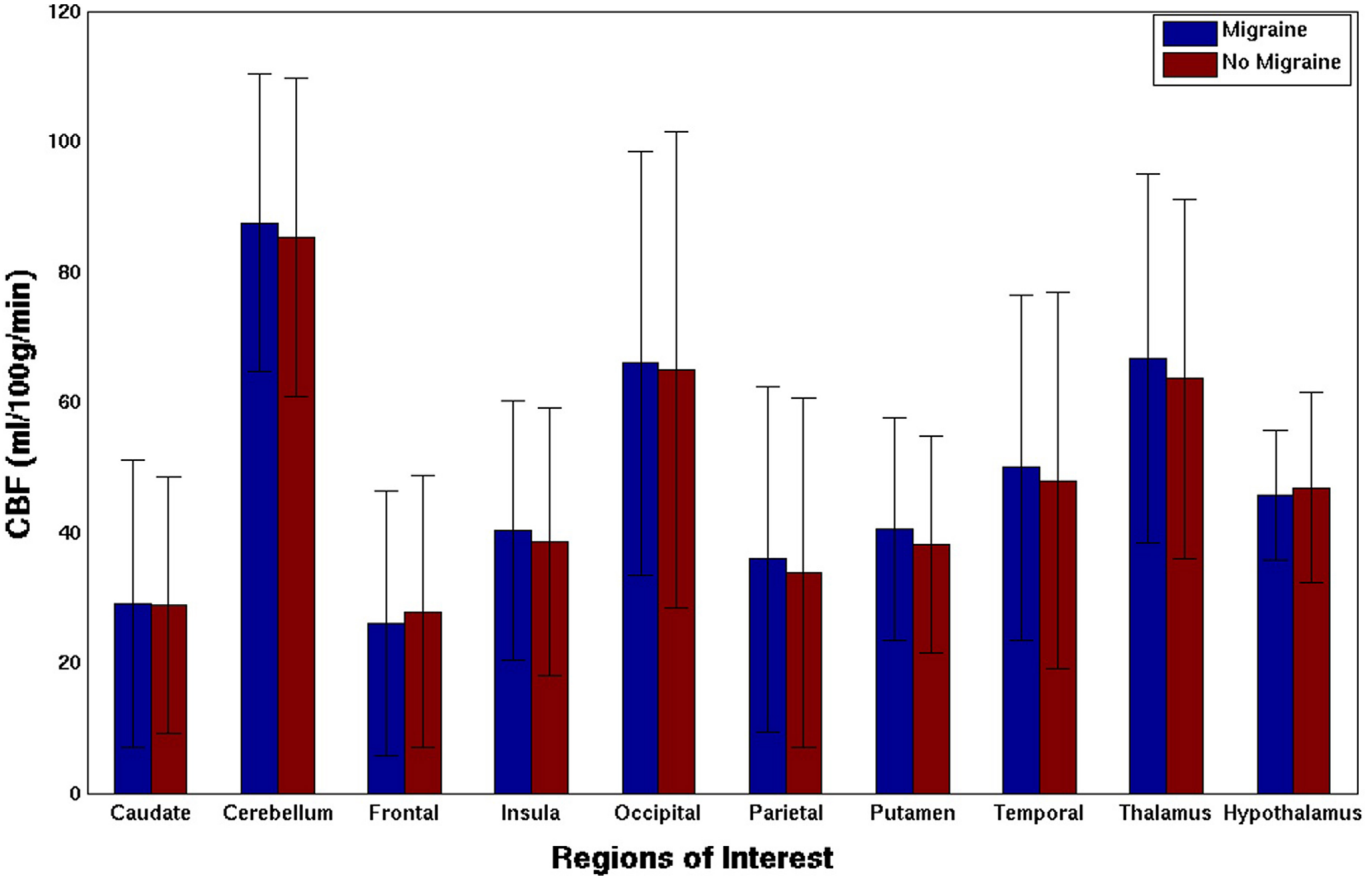
Cerebral blood flow (CBF) of different regions of interest during the migraine attack and attack-free status. Group average per session; Bars represent the mean, error bars the SD of the mean. No differences were found in any comparison (paired *T*-test, *p* n.s.).

The evaluated attack intensity was similar to usual attacks (paired *T*-Test 0.959, *p* = 0.357, n.s.) (Table 1). Average total CBF values were similar within and outside the attack (41.7 ± 8.8 versus 42.4 ± 6.2 ml/100 g/min, *p* = 0.589, n.s.). Perfusion weighted maps across subjects comparing migraine and non-migraine sessions are plotted for total CBF (Figure 1). No differences were found when comparing CBF of several ROI between sessions (Figure 2).

**Table 1:** Characterization of headache-free and attack variables.

Subjects	Headache-free		Migraine attack						
			Pain			Ass. symptoms			
	HIT-6	Total CBF	Total CBF	Duration	Intensity	Nausea	Photophobia	Phonophobia	Worse with P. effort
1	68	45.0	44.1	7.67	8.0	4.0	3.0	7.0	4.0
2	64	33.7	27.5	24.17	6.0	2.0	3.0	3.0	2.0
3	63	42.3	39.1	2.67	6.0	4.0	2.0	2.0	4.0
4	60	32.5	30.4	69.00	5.0	4.0	4.0	4.0	3.0
5	65	41.5	35.1	9.67	9.0	6.0	8.0	8.0	9.0
6	60	46.9	45.6	45.70	8.0	5.0	5.0	0	0
7	52	45.2	52.1	13.25	6.0	3.0	5.0	5.0	6.0
8	60	46.0	40.5	6.25	8.0	5.0	7.0	7.0	6.0
9	59	50.6	57.0	5.00	9.0	5.0	5.0	6.0	6.0
10	62	42.4	41.7	4.00	5.0	6.5	6.5	8.0	8.0
11	65	37.8	40.9	6.67	7.0	5.0	5.0	5.0	5.0
12	63	36.5	36.8	4.50	6.0	1.0	5.0	5.0	2.0
13	65	50.8	51.1	12.00	6.0	5.0	5.0	5.0	5.0
Av ± SD	62 ± 4	42.4 ± 6.2	41.7 ± 8.8	16.2 ± 19.7	6.8 ± 1.4	4.7 ± 1.5	4.9 ± 1.7	5.0 ± 2.3	4.6 ± 2.5

*HIT-6, headache impact test (in points); CBF, cerebral blood flow (in ml/100 g/min); Duration, pain duration up to the scan (in hours); Intensity, pain intensity upon entering the scan (0–10 VAS); Nausea, nausea intensity upon entering the scan (0–10 VAS); Photophobia, photophobia intensity upon entering the scan (0–10 VAS); Phonophobia, phonophobia intensity upon entering the scan (0–10 VAS); Worse with P. effort, intensity of pain aggravation by physical effort upon entering the scan (0–10 VAS); Av ± SD, average ± SD*.

### *Post Hoc* Analysis

Cerebral blood flow variation (ΔCBF%) represented an average −2.25 ± 10.6% of CBF in the attack relative to headache-free (ranging from −18.4 to 15.3%). ΔCBF average value was −0.72 ± 4.46 ml/100 g/min, ranging from −6.40 to 6.90. Multiple linear regression failed to identify any independent variable influencing ΔCBF (Table 2). Due to the wide variation of scan timing and the range of ΔCBF values, patients evaluated early (≤5 h, *n* = 4) and late (>5 h) into the attack and patients having positive (migraine attack > headache-free, *n* = 5) and negative ΔCBF (headache-free > migraine attack) were compared; no differences were identified (Tables 3 and 4).

**Table 2:** Multiple linear regression to evaluate the effect of clinical (independent) variables on ΔCBF (dependent variable).

	ΔCBF (dependent variable)	
Independent variables	Unstandardized beta coefficient ± SE	*p*
**Population-related variables**		
Age (years)	−0.270 ± 0.203	0.232
Literacy (years)	0.163 ± 0.403	0.701
Disease Duration (years)	0.255 ± 0.131	0.100
Headache impact test-6 score	−0.708 ± 0.346	0.087
Time between evaluations (days)	0.023 ± 0.023	0.351
**Attack-related variables**		
Attack duration (h)	−0.027 ± 0.141	0.854
Pain intensity [Visual Analog Scale (VAS)]	0.076 ± 2.416	0.976
Nausea intensity (VAS)	−0.377 ± 2.462	0.884
Photophobia intensity (VAS)	−0.307 ± 1.662	0.861
Phonophobia intensity (VAS)	−0.317 ± 1.666	0.857
Worsening with P. effort (VAS)	0.490 ± 1.967	0.813

*p = significance level*.

**Table 3:** Comparison of clinical variables of patients scanned early (≤5 h) and late (>5 h) into the attack.

	Early attack (≤5 h) *N* = 4	Late attack (>5 h) *N* = 9	*p*
	Mean ± SD	Mean ± SD	
Age (years)	33.0 ± 7.6	36.9 ± 7.4	0.402
Literacy (years)	16.2 ± 3.3	15.1 ± 3.4	0.583
Disease duration (years)	22.2 ± 11.1	22.9 ± 10.5	0.923
Headache impact test-6 score	61.8 ± 1.9	62.1 ± 4.7	0.888
Pain intensity [Visual Analog Scale (VAS)]	6.5 ± 1.7	7.0 ± 1.3	0.577
Nausea intensity (VAS)	4.1 ± 2.3	4.3 ± 1.2	0.833
Photophobia intensity (VAS)	4.6 ± 1.9	5.0 ± 1.6	0.724
Phonophobia intensity (VAS)	5.2 ± 2.5	4.9 ± 2.4	0.810
Worsening with P. effort (VAS)	5.0 ± 2.6	4.4 ± 2.6	0.729
Attack total cerebral blood flow (CBF)	44.3 ± 11.1	40.8 ± 8.5	0.578
ΔCBF	1.2 ± 4.8	−1.3 ± 4.4	0.425

*p = significance level*.

**Table 4:** Comparison of clinical variables of patients with negative and positive ΔCBF.

	ΔCBF < 0 (headache-free > attack) *N* = 7	ΔCBF > 0 (headache-free < attack) *N* = 5	*p*
	Mean ± SD	Mean ± SD	
Age (years)	35.4 ± 6.0	37.8 ± 9.1	0.597
Literacy (years)	15.3 ± 3.3	15.6 ± 3.9	0.883
Disease duration (years)	18.0 ± 8.7	30.0 ± 9.6	0.047
Headache impact test-6 score	62.0 ± 3.1	60.8 ± 5.5	0.424
Pain intensity [Visual Analog Scale (VAS)]	7.1 ± 1.5	6.8 ± 1.3	0.695
Nausea intensity (VAS)	4.3 ± 1.2	3.8 ± 1.8	0.590
Photophobia intensity (VAS)	4.6 ± 2.2	5.0 ± 0.0	0.629
Phonophobia intensity (VAS)	4.4 ± 3.0	5.2 ± 0.4	0.525
Worsening with P. effort (VAS)	4.0 ± 2.9	4.8 ± 1.6	0.592
Headache-free total cerebral blood flow (CBF)	41.1 ± 5.8	44.2 ± 6.8	0.423
Attack total CBF	37.5 ± 6.8	47.6 ± 8.4	0.044
ΔCBF	−3.6 ± 2.3	3.4 ± 3.2	0.001
ΔCBF%	−9.2 ± 6.2	7.5 ± 5.0	0.001

*p = significance level*.

## Discussion

Our study did not show any variation of global or regional CBF during attacks of migraine without aura in female migraine patients compared with their headache-free status, supporting some of the existing data (2, 5, 6, 9).

The initial cerebral perfusion studies in migraine used ^133^Xe, yet they have several serious limitations—used small samples or a few cases, had frail designs, some were not controlled and the techniques were invasive (sometimes two techniques were used in different subjects), were lengthy, difficult to reproduce, and had low accuracy. Some studied headache in attacks *with aura* revealing hyperperfusion in the headache phase (1, 19–21) or, more precisely, CBF increases occurring after the end of the aura, yet not clearly timed nor associated with the headache itself (22, 23). The distinction of aura and headache in this studies was arbitrary; in practice, these phenomena often overlap (24), which makes it very difficult to establish any kind of association.

A few studies of ^133^Xe also included patients with attacks *without aura*, with discrepant results, some revealing increase of mean CBF (12, 25–28), whereas most did not (6, 9, 29), and in some single cases pointed out revealing either global hyperperfusion or focal hypoperfusion in different regions (5, 6, 9, 10). Furthermore, in the subjects in whom some perfusion differences were found, those were below the range found in aura (7) or incongruent (10). Differences of results or inability to document changes could also be associated with patient selection, scan timing, and measurement reproducibility, besides the technical limitations. In view of all these limitations, information drawn from these earlier studies is neither reliable nor consensual.

Only three more recent studies have addressed some of these limitations by studying spontaneously occurring migraine *without aura* attacks controlled by an headache-free scan (2, 7, 13) and choosing more recent, fast, reproducible methods with good spatial resolution, although both requiring intravenous injection of contrast material. One ${\text{H}}_{2}^{15}\text{O}$ PET study included 13 patients with spontaneous unabated migraine without aura beginning in the previous 24 h that had not been treated with narcotics or vasoconstrictors, yet NSAIDs were allowed if taken >4 h before the scan; an at least 48 h headache-free scan was scheduled at the patient convenience. Only nine patients completed the study, having been scanned on average 13.3 h (range 3.8–24.5 h) after headache onset. A mean global 9.9% decrease in CBF (6.95 ml/min/100 g, *p* = 0.028) and of 5.2% of CBV (0.22 ml/100 g, *p* = 0.047) was observed during headache compared with the headache-free status and unrelated to the timing of the scan. Interestingly, oxygen metabolism and oxygen extraction fractions did not differ during headache compared with headache-free scans (7). The second ${\text{H}}_{2}^{15}\text{O}$ PET study included seven patients with spontaneous untreated migraine without aura scanned earlier, on average 3 h 08 min into their pain and later at around 6 h into the attack, after pain-induced relief with sumatriptan. A third headache-free scan was done 15–60 days later. A mean 10.34% bilateral regional relative decrease of adjusted rCBF was found in the occipital, posterior temporal, and parietal cortex of five out of the seven patients. After pain relief by sumatriptan, these findings were aggravated, as the adjusted rCBF decrease was 12.32% and hypoperfusion was also identified in the frontal areas, compared with the non-pharmacological-induced headache-free status (13).

A third study used PWI-MRI and included 13 spontaneously occurring and untreated migraine without aura attacks scanned on average 4 h 30 ± 2 h 50 (from 1 to 11 h) into the headache and again after at least a week headache-free. No changes were observed between scans in regional (occipital, mesencephalon, cingulum, and thalamus) CBF, CBV, and Mean Transit Time (2).

The use of ASL-MRI is a strength of our study, as it was not used before in this context and could help to enlighten the inconsistency found in previous studies. It is a highly reproducible and accurate technique (30), allows quantitative measurement of CBF, and is most sensitive to the perfusion changes at the capillary level (14) without the need of contrast nor radiation. The ASL-MRI major limitation (the arterial arrival time differences) is not an issue in this population (31).

Our findings give some robustness to the PWI-MRI study (2), supporting that no significant perfusion changes occur in the brain microvasculature during attacks of migraine without aura, contrary to the findings of the PET studies (7, 13) where some changes were documented.

Designs and sample sizes of the available studies are very similar, using spontaneous and untreated attacks. In our study, we documented that the scanned attack was representative of usual attacks, occurring in a high-impact episodic migraine sample population free of preventive treatment. Additionally, all our subjects had long-lasting (on average 22 years) migraine without aura, being unlikely to develop aura in the future, so the issue of different underlying neurobiology is unlikely to apply. We did not impose any limit to the duration of pain in our study in order to reach a moderate pain intensity and ensure that the scan evaluated a fully symptomatic attack (32), although bearing in mind that variability occurs between patients and attacks and that no clinical marker exists to identify attack phases. As a minimum pain intensity was required, we managed to study a representative attack that had an average intensity of 6.8 ± 1.4 on a 0–10 VAS. Our scans were done, as a consequence, on average later than two of the previous studies (2, 13)—at 16.2 ± 19.7 h, in a time frame comparable to the first PET study (7). Due to the design, the scans were acquired over a wide range of time frames into the attack, from 4 to 69 h. Yet, we were unable to find changes in our subgroup of four patients scanned early (≤5 h), and this subgroup was in all aspects similar to the group of nine patients scanned later, suggesting that timing of acquisition had no influence on results.

In a *post hoc* analysis, we separately evaluated the subgroup of patients in whom a decrease in CBF was documented during the attack compared with headache-free status (*N* = 7); in this group, the average decrease was of 9.2% (3.6 ± 2.3 ml/min/100 g, *p* n.s.), a percentage value close to that of one of the PET studies, yet half of its absolute value (7); even in this PET study, the changes found were close to non-significance and difficult to value with such a small sample size. We were unable to identify absolute values of CBF decrease in the second PET study (13). The decrease in CBF documented by PET is more likely to occur in the dependency of large vessels (30) and in one study (7) was accompanied by a normal to increased oxygen metabolism and extraction, suggesting the stability of tissue metabolic rate despite the reduction of the blood flow. The absence of abnormalities using ASL and PWI-MRI (2), which are superior in detecting microvascular variations and therefore more likely to detect changes at neuronal level (30), is congruent with normal tissue oxygen extraction.

One of our study’s limitations is the risk that the potential low magnitude of perfusion changes accepted to occur in this setting could have fallen in-between the limits of the techniques’ reproducibility (<10% CBF change) (33). Additionally, we have to consider all the potential biases that influence CBF, such as caffeine intake (34), tobacco smoking (35), hematocrit (36), and the normal circadian variation of CBF values (37), as it was not possible to perform the scans of both studies in each patient in the same schedule. It has been suggested that a cohort of less than 15 patients would be enough to obtain valid results (38); however, if we consider migraine heterogeneity, our sample size may not be sufficient to identify patients subgroups, although being one of the largest published. *Post hoc* analyses performed in this study impose further limitations of results’ interpretation.

In conclusion, our study supports that significant capillary perfusion changes are not expected to occur during migraine without aura attacks.

## Ethics Statement

This study was carried out in accordance with the recommendations of Comissão de Ética para a Investigação Clínica (CEIC) with written informed consent from all subjects, in accordance to the Declaration of Helsinki. The protocol was approved by the Hospital da Luz Ethics Committee.

## Author Contributions

RG-G has contributed to study concept and design, study coordination and supervision, data acquisition, statistical analysis, interpretation of the data, drafting and revising the manuscript, and obtaining funding. JP and PF have contributed to data acquisition and data analysis, interpretation of the data, and drafting and revising the manuscript. PV has contributed to study concept and design, study coordination and supervision, data acquisition, interpretation of the data, drafting and revising the manuscript, and obtaining funding. IM has contributed to study concept and design, interpretation of the data, revising the manuscript, and obtaining funding.

## Conflict of Interest Statement

The authors declare that the research was conducted in the absence of any commercial or financial relationships that could be construed as a potential conflict of interest.

## Abbreviations

ASL, arterial spin labeling; CBF, cerebral blood flow; PWI, perfusion weighted imaging; ICHD, International Classification of Headache disorders; VAS, Visual Analog Scale; HIT, headache impact test.
